# Dynamic changes and prognostic utility of procalcitonin, D-dimer, and lactate dehydrogenase in patients with sepsis and septic shock

**DOI:** 10.3389/fmed.2026.1771448

**Published:** 2026-03-02

**Authors:** Qiuxia Ge, Weijuan Song, Hongmei Ding, Haojie Wu, Zhen Ren

**Affiliations:** 1 Department of Laboratory Medicine, The First Affiliated Hospital of Nanjing Medical University, Nanjing, China; 2 Branch of National Clinical Research Center for Laboratory Medicine, Nanjing, China

**Keywords:** D-dimer, lactate dehydrogenase, PCT, sepsis, septic shock

## Abstract

**Objective:**

To investigate whether serum levels of procalcitonin (PCT), D-dimer (D-D), and lactate dehydrogenase (LDH) are associated with disease severity and differ between patients with sepsis with favorable versus poor in-hospital outcomes.

**Methods:**

This retrospective cohort study included 171 patients with sepsis. Patients were stratified into a septic shock group (*n* = 49) and a non-shock sepsis group (*n* = 122) to assess disease severity and further categorized into an improved group (*n* = 127) and a poor prognosis group (*n* = 44) based on discharge outcomes to evaluate prognostic value. Univariate and multivariate logistic regression analyses were performed to identify independent factors associated with septic shock. Dynamic trajectories of PCT, D-D, and LDH levels during hospitalization were also analyzed.

**Results:**

On admission, levels of PCT, D-D, and LDH were significantly higher in the septic shock group than in the non-shock sepsis group (all *p <* 0.01). In the multivariable model, elevated levels of PCT (OR = 1.015, 95% CI: 1.002–1.028), D-D (OR = 1.087, 95% CI: 1.010–1.170), and LDH (OR = 1.265, 95% CI: 1.016–1.576) were independently associated with an increased likelihood of septic shock. Patients with a poor prognosis exhibited persistently elevated levels of all three biomarkers throughout hospitalization, whereas these levels decreased significantly in the improved group.

**Conclusion:**

PCT, D-D, and LDH are valuable biomarkers for stratifying disease severity and predicting clinical outcomes in patients with sepsis. Admission levels of these biomarkers were independently associated with the development of septic shock, and their dynamic changes provided additional prognostic information.

## 1 Introduction

Sepsis, a life-threatening organ dysfunction resulting from a dysregulated host response to infection, remains a leading cause of morbidity and mortality in intensive care units worldwide (1–3). Its most severe clinical manifestation, septic shock, is characterized by profound circulatory and cellular/metabolic dysfunction and is associated with alarmingly high mortality (4, 5). The pathophysiological complexity of sepsis involves a dysregulated interplay among widespread inflammation, immune suppression, and coagulation dysfunction, thereby rendering early and accurate assessment of disease severity and prognosis critically important for guiding therapeutic decisions.

The current diagnostic paradigm for sepsis relies primarily on clinical criteria and microbiological cultures. Although microbiological culture remains the gold standard for pathogen identification, its utility in guiding urgent clinical decision-making is limited by several inherent drawbacks, including prolonged turnaround times (typically 24–72 h) and suboptimal sensitivity (6, 7). This diagnostic and prognostic gap underscores the need for reliable and rapidly available biomarkers to complement clinical judgment. Although numerous biomarkers have been investigated, no single biomarker adequately captures the multifaceted nature of sepsis. Consequently, a panel of biomarkers reflecting distinct pathological axes may offer superior clinical utility (8).

This study focuses on the combined analysis of procalcitonin (PCT), D-dimer (D-D), and lactate dehydrogenase (LDH), leveraging their distinct yet complementary pathophysiological roles in sepsis. PCT is a well-established biomarker of systemic bacterial infection and the associated inflammatory response, and its kinetic profile is useful for both diagnosis and monitoring therapeutic efficacy (9). D-D, a marker of activated coagulation and fibrinolysis, reflects the severity of sepsis-associated coagulopathy, a major contributor to organ dysfunction and mortality (10). LDH, which is released following cellular injury, serves as a non-specific marker of tissue hypoxia and cellular damage, providing an indicator of downstream effects of shock and microcirculatory dysfunction in sepsis (11).

While each biomarker provides clinically relevant information when considered individually, their combined application addresses three core pathophysiological components of sepsis: inflammation (PCT), coagulopathy (D-D), and cellular injury (LDH). We hypothesize that this biomarker panel will enable a more comprehensive assessment than any single biomarker alone. Specifically, we propose two hypotheses: (1) elevated levels of all three biomarkers on admission will more accurately identify patients at high risk of progression to septic shock, and (2) dynamic changes in these biomarkers during hospitalization will provide improved prognostic stratification of clinical outcomes. Accordingly, this study aims to investigate the independent and combined associations of PCT, D-D, and LDH levels with sepsis severity and clinical outcomes, with the goal of evaluating a pragmatic multi-marker panel to support clinical assessment.

## 2 Materials and methods

### 2.1 General information

This retrospective observational study included 171 patients with sepsis who were admitted to the First Affiliated Hospital of Nanjing Medical University between September 2020 and June 2025. Based on the 2021 Surviving Sepsis Campaign Guidelines for the Management of Sepsis and Septic Shock (12), patients were stratified into a non-shock sepsis shock group (*n* = 122) and a septic shock group (*n* = 49). According to discharge outcomes, patients were further categorized into a clinically improved group (*n* = 127) and a poor prognosis group (*n* = 44).

The clinically improved group comprised patients who were discharged with a diagnosis of “cured” or “improved,” whereas the poor prognosis group comprised the following two categories: (1) In-hospital mortality: patients who died from any cause during the index hospitalization. (2) Discharge against medical advice due to **critical** illness: patients whose family members requested discharge because of the critical condition and extremely poor prognosis, and who met at least two of the following objective criteria:

- transfer to the intensive care unit (ICU) for advanced life support;
- requirement for mechanical ventilation to provide respiratory support;
- presence of multiple organ dysfunction or failure, defined as involvement of two or more organ systems, each with an increase of ≥ 2 points in the Sequential Organ Failure Assessment (SOFA) score;
- a clinical assessment by the attending physician indicating an end-stage condition, leading the family to forego active treatment.

We applied the 2016 Sepsis-3 definitions using explicit, data-driven criteria derived from the hospital’s electronic medical records.

1. Sepsis: suspected infection plus acute organ dysfunction, defined as an increase in the SOFA score of ≥ 2 points. Suspected Infection (“time zero”) was defined as the first instance in which a patient received intravenous antibiotics and had a body fluid culture ordered within a 24-h period. For SOFA score calculation: the worst values within 24 h before and after “time zero” were used.

- Respiration: the SpO₂/FiO₂ ratio was used when arterial blood gas measurements were unavailable.
- Coagulation, liver, renal and central nervous system (GCS): the lowest platelet count and the highest bilirubin or creatinine values were used, and GCS scores were obtained from nursing records.
- Cardiovascular: assessment was based on mean arterial pressure and vasopressor use.
  1. Septic shock: patients meeting the sepsis criteria above and fulfilling both of the following conditions within 24 h after “time zero”:

- Vasopressor requirement: receipt of any continuous vasopressor infusion (e.g., norepinephrine or epinephrine) to maintain blood pressure.
- Hyperlactatemia: a serum lactate level > 2.0 mmol/L measured within 6 h after “time zero.”

*Inclusion criteria:*

Patients were included if they met all of the following criteria:

1. diagnosed with sepsis or septic shock according to the 2021 Surviving Sepsis Campaign Guidelines for the Management of Sepsis and Septic Shock (12);
2. aged 18 years or older;
3. availability of complete clinical records.

*Exclusion criteria:*

Patients were excluded if any of the following criteria were met:

1. known hematological diseases;
2. a history of malignant tumors;
3. transfer to another facility or deceased within 24 h of admission.

### 2.2 Ethical approval

The study protocol was approved by the Ethics Committee of the First Affiliated Hospital of Nanjing Medical University (2024-SR-136). This study protocol complied with the principles of the Declaration of Helsinki.

### 2.3 Data collection

Demographic and clinical characteristics, including sex, age, primary site of infection, and identified pathogens, were extracted from electronic medical records. Laboratory data were collected at two predefined time points. On admission, defined as the first available measurement obtained within 24 h of hospital admission, routine blood tests and inflammatory markers, including PCT, D-D, and LDH, were collected.

To evaluate biomarker dynamics, levels of PCT, D-D, and LDH were also measured in fasting venous blood samples obtained within 24 h before discharge in both the clinically improved and poor prognosis groups. All laboratory tests were performed in the hospital’s clinical laboratory in accordance with standardized operating procedures.

PCT was measured using an electrochemiluminescence immunoassay on a Roche Cobas e 801 analyzer. Results were reported in ng/mL, with a reference range of <0.05 ng/mL. D-D was measured using an immunoturbidimetric assay on a Sysmex CS-5100 coagulation analyzer. Results were reported in mg/L, with a reference range of <0.55 mg/L. LDH was measured using the rate method on a Beckman Coulter AU5800 biochemical analyzer. Results were reported in U/L, with a reference range of 125–243 U/L. All assays were conducted in accordance with the manufacturer’s reagent instructions and the laboratory’s internal quality control protocols.

### 2.4 Statistical analysis

Statistical analyses were performed using SPSS version 21.0 (IBM Corp.), and figures were generated using GraphPad Prism version 9.0. Continuous variables with non-normal distributions were summarized as medians with interquartile ranges (IQRs). Categorical variables were compared using the chi-square test.

Given the potential for skewed distributions and outliers in laboratory measurements, non-parametric tests were used for group comparisons. Specifically, the Wilcoxon rank-sum test (Mann–Whitney U test) was used to compare two independent groups (e.g., septic shock vs. non-shock sepsis). For paired comparisons (e.g., admission vs. discharge levels), the Wilcoxon signed-rank test was applied. Outliers were identified using Tukey’s method and were retained in the primary analysis. The robustness of the main findings was further assessed through sensitivity analyses performed after excluding these outliers.

Independent factors associated with septic shock were identified using multivariate binary logistic regression. To improve the interpretability of effect estimates, LDH was log-transformed and rescaled. All tests were two tailed, and a *p <* 0.05 was considered statistically significant.

## 3 Results

### 3.1 Comparison of baseline characteristics between groups

Baseline demographic characteristics, including age and sex, were comparable between the non-shock sepsis group and the septic shock group (*p* > 0.05). The distribution of primary sites of infection and identified pathogens is summarized in Table 1.

**Table 1:** Comparison of baseline characteristics between groups.

Indicator	Indicator non-shock sepsis group (*n* = 122)	Septic shock group (*n* = 49)	*χ*^2^/*Z*	*p*-value
Age (y)	61.00 (53.00, 71.00)	59.00 (51.00, 68)	−0.919	0.358
Sex [*n* (%)]				
Male	79 (64.75)	39 (79.59)	3.599^*^	0.058
Female	43 (35.25)	10 (20.41)		
Pathogen [*n* (%)]				
*Escherichia coli*	11 (9.02)	11 (22.45)		
*Klebsiella pneumoniae*	25 (20.49)	20 (40.82)		
*Staphylococcus aureus*	16 (13.11)	2 (4.08)		
*Enterococcus faecium*	14 (11.48)	4 (8.16)		
*Pseudomonas aeruginosa*	8 (6.56)	0 (0)		
*Acinetobacter baumannii*	7 (5.74)	4 (8.16)		
*Candida albicans*	15 (12.30)	6 (12.24)		
Primary disease [*n* (%)]				
Pulmonary infection	26 (21.31)	10 (20.41)		
Abdominal infection	25 (20.49)	23 (46.94)		
Urinary tract infection	26 (21.31)	8 (16.33)		
Skin/soft tissue infection	11 (9.02)	3 (6.12)		

*Denotes *χ*^2^ value.

### 3.2 Comparison of laboratory indicators between groups

Levels of the key study biomarkers PCT, D-D, and LDH, were significantly elevated in the septic shock group compared with the non-shock sepsis group (all *p <* 0.05). Detailed comparisons of all laboratory parameters, including aspartate aminotransferase (AST), are presented in Table 2.

**Table 2:** Comparison of laboratory indicators between groups.

Indicator	Indicator non-shock sepsis group (*n* = 122)	Septic shock group (*n* = 49)	*Z*	*p*-value
WBC (×10^9^/L)	10.53 (7.29, 16.16)	12.61 (6.77, 21.26)	−1.158	0.247
RBC (×10^12^/L)	3.60 (3.01,0.4.17)	3.64 (3.07, 4.32)	−0.670	0.503
HB (g/L)	107.00 (89.75, 123.25)	113.00 (95.00, 129.00)	−1.003	0.316
PDW (%)	15.90 (13.00, 16.40)	16.00 (12.20, 16.90)	−0.807	0.420
CRP (mg/L)	95.67 (58.20, 173.62)	90.00 (62.30, 223.03)	−0.535	0.593
PCT (ng/mL)	3.03 (0.30, 14.36)	10.64 (1.57, 64.84)	−3.771	0.000
FIB (g/L)	4.92 (3.83, 6.67)	4.84 (2.67, 6.51)	−0.765	0.444
D-D (mg/L)	2.30 (1.16, 4.28)	4.43 (1.66, 8.73)	−2.903	0.004
ALT (U/L)	33.00 (16.00, 62.40)	35.40 (20.80, 86.85)	−1.344	0.179
AST (U/L)	32.20 (22.50, 63.70)	58.50 (30.45, 120.70)	−3.097	0.002
GGT (U/L)	70.00 (29.10, 121.75)	65.00 (24.00, 166.00)	−0.401	0.688
LDH (U/L)	263.00 (208.00, 344.00)	349.00 (288.00, 495.00)	−4.134	0.000

### 3.3 Factors associated with the development of septic shock

Univariate and multivariable logistic regression analyses indicated that elevated levels of PCT, D-D, and LDH were significantly associated with the occurrence of septic shock after adjustment for potential confounders (*p <* 0.05). The magnitude of these associations is presented in Tables 3, 4.

**Table 3:** Univariate logistic regression analysis.

Variable	*β*	*SE*	Wald *χ^2^*	*p*-value	*OR*	95% *CI*
PCT (ng/mL)	0.022	0.006	13.814	0.000	1.022	1.010–1.034
D-D (mg/L)	0.126	0.040	9.988	0.002	1.134	1.049–1.226
AST (U/L)	0.005	0.002	5.858	0.016	1.005	1.001–1.009
Log (LDH)	0.335	0.092	13.334	0.000	1.397	1.168–1.672

**Table 4:** Multivariate logistic regression analysis.

Variable	*β*	*SE*	Wald *χ^2^*	*p*-value	*OR*	95% CI
PCT (ng/mL)	0.015	0.006	5.269	0.022	1.015	1.002–1.028
D-D (mg/L)	0.084	0.038	4.953	0.026	1.087	1.010–1.170
Log (LDH)	0.235	0.112	4.418	0.036	1.265	1.016–1.576

Based on the logistic regression analyses, a combined predictive model for sepsis prognosis was derived using the three biomarkers PCT, D-D, and LDH. The predictive equation for sepsis prognosis was defined as follows:


p=−7.556+0.015×PCT+0.235×log(LDH)+0.084×D−D

### 3.4 Comparison of biomarker levels between admission and discharge

**Figure 1:**
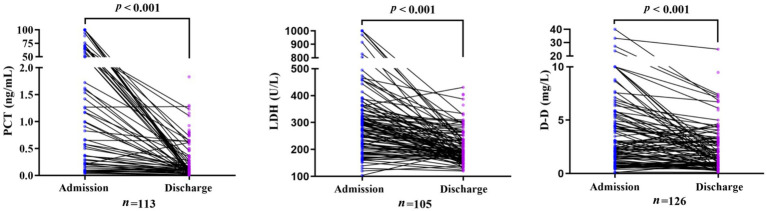
Change in PCT, LDH, and D-D levels in the clinically improved group from admission to discharge.

**Figure 2:**
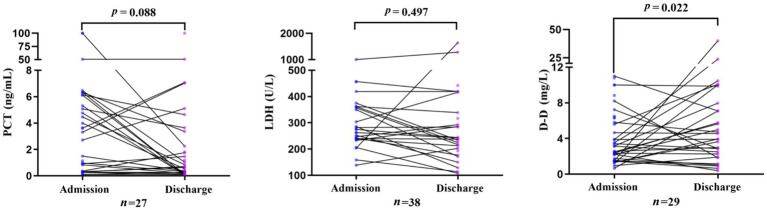
Change in PCT, LDH, and D-D levels in the poor prognosis group from admission to discharge.

In the clinically improved group, levels of PCT, D-D, and LDH decreased significantly from admission to discharge (all *p* < 0.05). In contrast, in the poor prognosis group, D-D levels increased significantly at discharge (*p <* 0.05), whereas PCT and LDH levels showed no significant change during hospitalization (Figures 1, 2).

In the paired analyses, the valid sample sizes for PCT, LDH, and D-D in the clinically improved group were 113, 105, and 126, respectively. The corresponding sample sizes in the poor prognosis group were 27, 38, and 29, respectively.

## 4 Discussion

Sepsis remains a major global health challenge and is associated with substantial morbidity and mortality. Timely and accurate assessment is critical, as progression to septic shock markedly increases the risk of multiple organ dysfunction and death (13–15). However, the early clinical manifestations of sepsis are often non-specific, and definitive microbiological diagnosis via culture is invariably delayed, thereby creating a therapeutic window during which rapid clinical assessment and severity stratification are pivotal for disease diagnosis and management (16, 17). This limitation underscores the need for objective and readily available biomarkers to complement clinical judgment. Accordingly, this study aimed to identify laboratory-based factors associated with septic shock and to evaluate their utility in assessing disease severity and prognosis in patients with sepsis.

This study demonstrates that levels of PCT, D-D, and LDH, either individually or in combination, are significantly associated with disease severity and clinical outcomes in patients with sepsis. The key findings are twofold: first, elevated levels of PCT, D-D, and LDH on admission were independently associated with the development of septic shock; second, serial monitoring of these biomarkers revealed distinct dynamic patterns that were strongly correlate with final clinical outcomes.

These results corroborate and extend the existing literature on individual biomarkers. The role of PCT as an indicator of systemic bacterial infection and inflammatory burden is well established (18, 19). This study reinforces this role by quantifying the association between PCT levels and disease severity, thereby its utility not only as a diagnostic marker but also as an indicator of the magnitude of the host’s dysregulated inflammatory response. Similarly, elevation of D-D is a recognized hallmark of sepsis-associated coagulopathy, a key contributor to microvascular thrombosis and end-organ damage (20, 21). The present analysis strengthens this association by demonstrating that elevated D-D levels are independently associated with the development of septic shock. Notably, D-D levels exhibited a divergent temporal trajectory, increasing significantly in patients with poor outcomes. This dynamic behavior suggests that D-D may serve as a sensitive indicator of ongoing, potentially treatment-resistant coagulopathy, providing prognostic information beyond a single baseline measurement.

Although LDH is a relatively non-specific marker of cellular injury, its pronounced elevation in patients with septic shock is consistent with the pathophysiology of global tissue hypoxia and cellular necrosis (23–25). The absence of a significant decline in LDH levels among patients with poor outcomes further underscores its value as a persistent indicator of cellular damage, complementing the inflammatory and coagulation profiles reflected by PCT and D-D. The elevation of aspartate aminotransferase (AST) observed in the septic shock group likely reflects concomitant hepatic or cardiac injury (22). However, the contribution of AST to sepsis pathophysiology appears less direct than that of the primary biomarker triad, which collectively reflects the core perturbations of inflammation (PCT), coagulation (D-D), and cellular injury (LDH).

The principle clinical strength of this study lies in the evaluation of the combined and dynamic use of these biomarkers. Individually, each biomarker provides insight into a specific aspect of sepsis pathophysiology; collectively, they enable a more comprehensive assessment of patient status. The consistent decline of all three biomarkers in patients with favorable outcomes, compared with their persistent elevation or increase in patients with poor outcomes, reflects differential responses to therapy. These findings support a shift from static, admission-only biomarker assessment toward a dynamic monitoring strategy, in which longitudinal trends may provide earlier and more reliable prognostic information than absolute values obtained at a single time point.

## 5 Limitations

Several limitations should be considered when interpreting the findings of this study. First, as a single-center, retrospective study, the results may be influenced by inherent selection bias. The included patients represent a specific local population and healthcare environment, which may limit the generalizability of the findings to other institutions or regions with differing patient demographics, clinical practices, or resource availability.

Second, the study period spanned from 2020 to 2025, during which clinical protocols, including those for sepsis management and biomarker testing, may have evolved. Third, the classification of patient outcomes, particularly discharge status (e.g., “improved” vs. “poor prognosis”), may be subject to variability in clinical decision-making. Discharge timing and criteria may vary among treating physicians, potentially introducing misclassification bias.

Fourth, a primary limitation of this study lies in its retrospective design, which precluded the ability to obtain and adjust for several known and clinically important prognostic confounders (e.g., age, severity of underlying illness, such as the SOFA score, and specific ICU interventions). Consequently, the associations observed between the biomarkers (PCT, D-D, and LDH) and clinical outcomes may be subject to residual confounding. These findings should therefore be interpreted as preliminary signals of association rather than as evidence establishing these biomarkers as independent predictive factors. Future multicenter prospective studies with standardized protocols are needed to validate these findings and to enhance their external validity.

## 6 Conclusion

In conclusion, this study demonstrates that levels of PCT, D-D, and LDH are significantly associated with disease severity and clinical outcomes, highlighting their potential utility in the early assessment of patients with sepsis. More importantly, serial measurements of this biomarker triad reveal dynamic patterns that are strongly associated with clinical outcomes, providing a practical tool for monitoring treatment response. Incorporating this readily available biomarker panel into routine clinical practice may facilitate earlier identification of high-risk patients, guide the intensity of therapeutic interventions, and improve the personalization of sepsis management.

## Data Availability

The raw data supporting the conclusions of this article will be made available by the authors, without undue reservation.
